# The Moderating Role of Social Identity and Grit in the Association Between Parental Control and School Adjustment in Chinese Middle School Students

**DOI:** 10.3389/fpsyg.2020.00677

**Published:** 2020-04-24

**Authors:** Chunhua Ma, Yongfeng Ma, Xiaoyu Lan

**Affiliations:** ^1^College of Educational Science and Technology, Northwest Minzu University, Lanzhou, China; ^2^Department of Developmental Psychology and Socialisation, University of Padua, Padua, Italy

**Keywords:** school adjustment, social identity, grit, parental control, middle school students

## Abstract

Although the proliferation of empirical research has documented the association between parental control and school adjustment, findings of this linkage are still inconclusive. Moreover, fewer efforts have been made to address this association in middle school students. Guided by an ecological framework, the current study aimed to integrate the conflicting findings into a coherent body of knowledge, paying particular attention to two research purposes: (a) to examine the association between parental control and three objective indicators of school adjustment (social competence, academic grades, and peer acceptance) and (b) to explore whether this association was moderated by individual characteristics of social identity and grit. A total of 120 Chinese middle school students (42.5% females) aged between 13 and 15 years old were recruited for this study, and research data were gathered from multiple sources. To be specific, students were asked to complete a set of self-report questionnaires concerning parental control, social identity, and grit. Meanwhile, school-related social competence was rated by head teachers; academic grades were obtained from school records; and peer acceptance was assessed by sociometric nominations. The results from hierarchical regression analyses showed that parental control was negatively associated with academic grades. Moreover, when reporting higher levels of social identity, parental control was negatively related to social competence and peer acceptance for those students with lower levels of grit. Our findings suggest that parental control can dampen middle school students’ academic performance, and low levels of grit can magnify the detrimental effect of parental control on social competence and peer acceptance in middle school students who regard themselves as closely connected to social groups.

## Introduction

As described by a well-known Chinese proverb, “all pursuits are of low value; only studying the books is high (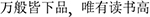; Yu and Suen, 2005, p. 18), Chinese culture highlights greater educational attainment. A successful school adjustment, encompassing excellent academic performance, socially appropriate behaviors, and positive peer relationships, is at the core of Chinese students’ development (Bond, 2010). Nevertheless, school adjustment is often challenging, especially when students enter middle school. During this period, the significant changes in biological, cognitive, and social relations often result in a high level of psychosocial and academic maladjustment, such as emotional-behavioral difficulties, poor academic grades, and worse peer relationships (Kaltiala-Heino et al., 2003; Wang and Dishion, 2012; Gregson et al., 2017). Given these challenges, further investigation into the correlates of school adjustment in middle school students is critical in order to provide some possible insight into designing and administrating effective intervention or prevention programs. In consideration with cultural orientations and empirical indications (e.g., Chen et al., 2019), in this study, we focused on three indicators of school adjustment: social competence (referring to a series of interpersonal skills and socially appropriate behaviors; Harter, 1982), academic grades, and peer acceptance (the number of classmates who nominate him or her among their three most-liked classmates; Chen et al., 2004).

Among possible correlates of school adjustment, it has been demonstrated that parents have a prominent role in shaping students’ adjustment to the school environment (Ratelle et al., 2017; Lan et al., 2019c). As a central part of the socialization process in many cultural contexts (Grolnick and Pomerantz, 2009), we focused on parental control in the current study. Although extensive studies have addressed parental control and its impact on students’ school adjustment in many cultural contexts (see a useful meta-analysis by Pinquart, 2016), China included (e.g., Chen et al., 2000; Xia et al., 2015), findings of this association remain somewhat inconclusive and inconsistent. Therefore, further investigation is imminent to shed light on this association and ascertain whether individual characteristics may explain the variance of this linkage.

In the current study, we took an ecological framework to address the correlates of (both contextual and individual factors) school adjustment (Bronfenbrenner, 1979). This is because school adjustment is an ecological phenomenon, which has been developed over time as a consequence of complex interactions across multiple contexts (Jacobson and Crockett, 2000). In view of this, the ecological framework provides a comprehensive understanding of the factors associated with school adjustment. Such a framework has been extensively applied to empirical research investigating the correlates of school-related outcomes (e.g., Jacobson and Crockett, 2000; Hong and Garbarino, 2012; Huang et al., 2013). Following this framework, students are embedded in an interactive set of contextual and personal systems. For instance, contextual factors (e.g., parental control) may interact with individual characteristics to explain the variance of school adjustment. Since individuals are nested in different layers of environments, it is more valuable and informative to investigate how multiple factors (across domains or contexts) jointly or interactively (instead of investigating the main effects individually) shape students’ adjustment (Zhou et al., 2012; Lan and Zhang, 2019).

Concerning individual characteristics, we focused on social identity (one’s knowledge of membership in a social group; Tajfel, 2010) and grit (continuous perseverance and volitional control; Duckworth et al., 2007). This is because these personal attributes have been found to buffer the adverse effect of environmental stressors on students’ school functioning in separate lines of research (e.g., Juvonen, 2006; Lan and Moscardino, 2019). Given this significance, to our knowledge, the role of social identity and grit in the association between parental control and school adjustment has not yet been addressed in one single investigation. Identifying whether these personal characteristics may explain the variance of the association between parental control and school adjustment is valuable. This is because these attributes are relatively malleable, and educators and practitioners would be able to use them to intervene in school-related activities.

Apart from these theoretical considerations, numerous studies investigating the association between parental control and school adjustment heavily rely on self-report assessments (although enabling large-scale data collection), which is potentially affected by common method bias and social desirability (Podsakoff et al., 2003). It is widely assumed that these biases may inflate the associations between variables assessed by self-reports. To potentially reduce this methodological weakness, we adopted a multiple source approach (i.e., gathering information from peer evaluations, teacher ratings, self-reports, and school records) to assess study variables in middle school students. Such an approach may help researchers to have a more holistic picture of students’ school adjustment and has successfully applied to many well-established empirical studies (e.g., Chen et al., 2019).

To briefly summarize, the current study, using an ecological framework, aimed to investigate the association of parental control with school adjustment among Chinese middle school students as well as examine whether social identity and grit may moderate this association. In the following sections, we conducted a literature review to summarize the possible associations of parental control, social identity, and grit with school adjustment in school-aged students. Although we endeavored to cover the relevant literature adequately and in an unbiased manner, the primary principle of this literature review tightly adhered to culture-specific perspectives, given that parental control is culturally variable concerning its normativeness, meanings, and potential consequences (Deater-Deckard et al., 2011). In a sense, converging evidence across Western and Eastern cultural contexts is challenging to collect (although valuable and informative), and different cultural backgrounds may shape the association between parental control and students’ school adjustment in a different fashion (Pomerantz and Wang, 2009). To have a more comprehensive understanding of the study associations, we did review the existing literature across distinct cultural contexts. However, we were cautious when generating specific hypotheses, doing so according to the empirical evidence from the Western cultural contexts.

### Parental Control and School Adjustment

Parental control refers to how parents force their children to meet demands, solve problems for them, and take a parental rather than child-oriented perspective (Grolnick, 2003). Indeed, parents’ exertion of control over children, as a central part of the socialization process, has been widely identified in many cultural contexts (Grolnick and Pomerantz, 2009). The majority of empirical findings from Western backgrounds has revealed that parental control is negatively associated with school indicators (Pinquart, 2016; Pinquart and Kauser, 2018). This is because individualist societies highlight self-orientation, personal development, and autonomy.

To be specific, parental control has been found to bear a negative association with students’ academic achievement (Kokkinos and Vlavianou, 2019). The authors explain that parental control can lead students to focus more on internal distress and adults’ approval rather than the learning process itself, which further hinders students’ learning and academic performance. Moreover, findings have indicated that parents who are more controlling may deprive their children of opportunities for regulating emotions and developing positive coping strategies, thereby resulting in worse social competence and negative peer interactions for their children (McDowell and Parke, 2005).

By contrast, in collectivistic cultures, such as that of China, children may react less negatively to parental control than their Western peers (Chen et al., 2000; Wang et al., 2007; Li et al., 2015). This pattern has been attributed to the fact that Chinese parents emphasize filial piety (how children treat their parents; see Yeh and Bedford, 2003), and children are socialized to meet their parents’ expectations and rules in line with Confucian values (Chao, 1994). As indicated by prior research (Chao, 1994; Shek, 2007), indigenous Chinese concepts of parental control—such as *sheng xin* (mature), *ting hua* (obedient), and *du cu* (urge)—are essential concepts to understand when it comes to Chinese parenting. Due to the critical role of family dignity and social order in the Chinese society, parents usually expect youth to be mature and obedient as well as urge them to meet parental expectations, such as achieving excellent school performances and bringing honor to the family (Shek, 2007). Given these cultural orientations, we operationalized and assessed parental control based on indigenous Chinese concepts to fully capture this notion in a specific cultural background.

According to empirical findings of Chinese students, it has been illustrated that parental control can significantly and positively predict middle school students’ social competence (Chen et al., 2000). Moreover, previous study has found that parental control has a conducive effect on middle school students’ academic grades (Wang et al., 2007), indicating that parents’ intrusion upon children’s autonomy and independence seems to positively impact academic performance. Additionally, research has shown that parental solicitation and restriction is harmless to middle school students’ relational peer experiences (Li et al., 2015).

Despite these conflicting results (as compared with the findings obtained from the Western contexts), in the past decades, China has witnessed dramatic modernization, urbanization, and related social changes. In a sense, Chinese parenting styles are potentially expected to be adjusted (Zhang et al., 2017). As documented by recent findings (e.g., Li and Lamb, 2015), Chinese parents now exhibit sizable warmth and autonomy support during parent–child interactions. Although parental control may be still considered one of the most common parenting styles in the Chinese society, it seems undeniable that parents have started to be more engaged and supportive of autonomy in daily child-rearing, and they often have to negotiate with their children in terms of personal goals and willingness (Li and Lamb, 2015; Lan et al., 2019b). Given these dramatic sociocultural and economic advances, we expected that parental control might have been negatively linked to students’ school adjustment (Hypothesis 1). Moreover, given the conflicting findings in the existing literature, it would be more meaningful to address whether individual characteristics of social identity and grit may explain the variance between parental control and school adjustment.

### The Moderating Role of Social Identity

Social identity refers to one’s knowledge of membership in a social group, together with the value and emotional significance that one has attached to that group membership (Tajfel, 2010). As indicated by prior research (Romera et al., 2016), students with negative self-concept (e.g., low self-esteem and self-efficacy) are more likely to avoid social interactions and social reinforcement, thus making it difficult for them to achieve adequate adjustment at school through instances such as interacting with classmates.

Moreover, research has shown that social identity is strongly influenced by cultural norms and values (Brewer and Yuki, 2007). In individualistic societies, the ties between individuals are relatively loose, as individuals are expected to attend to themselves and their immediate family. By contrast, in collectivist cultures, such as China, individuals are firmly integrated into a cohesive in-group community (e.g., immediate and extended families as well as classrooms and schools), in which group members protect each other in exchange for loyalty (Hofstede and McCrae, 2004). Individuals growing up in collectivistic societies are more prone to regarding themselves as closely connected to others, and individuals’ self-concept tend to be defined primarily based on relationships with other people within their in-groups (Liu et al., 2012), which has profound implications for individuals’ school adjustment (Markus and Kitayama, 2010). Although students in a collective context may show higher levels of social identity within their in-groups, little attention has been paid to individual differences in social identity, which may interact with contextual variables, such as parental control, to influence students’ school adjustment.

According to the existing evidence, Lan et al. (2019c) have demonstrated that interdependent self-construal can moderate the association between parental governing/training and school adjustment in Chinese students. To be precise, students with high parental governing/training and an interdependent self-construal exhibit high teacher-rated social competence and better academic grades. This finding indicates that the association of parental control with school adjustment may be contingent on how Chinese students regard themselves as closely connected to a social group. In this regard, students reporting stronger connectedness to a social group (e.g., a family) may be more likely to be influenced by parental control than those with lower levels of social identity, which may highlight the association between parental control and school adjustment (Hypothesis 2).

### The Moderating Role of Grit

Grit refers to continuous perseverance and sustained interests toward long-term goals (Duckworth et al., 2007; Duckworth and Quinn, 2009). More precisely, grit consists of two facets: perseverance of effort and consistency of interests. Higher levels of perseverance enable students to overcome obstacles and challenges through enduring personal efforts and determination, and consistency highlights the capacity to maintaining long-term aspirations by focusing on their interests and targeted assignments (Duckworth and Quinn, 2009; Lan, 2019; Cui and Lan, 2020). Although it is still a highly debated issue concerning the differential roles of these two dimensions on students’ development, findings from a recent review (Credé et al., 2017) and the validation of the grit scale in a collective society (Datu et al., 2016) have supported the assertion that the construct of grit mainly focuses on perseverance of effort instead of consistency of interests. Moreover, perseverance and diligence are highly emphasized in the context of Chinese culture (Li et al., 2018; Lan et al., 2019b), and a persistent display of willpower in the face of difficulty is often regarded as a valuable individual characteristic. Given these empirical and cultural considerations, the current study explored the moderating role of grit in the association between parental control and school adjustment by focusing on perseverance only.

According to empirical research, grit has been demonstrated to be a strong predictor for students’ academic achievement in many cultural contexts (e.g., Muenks et al., 2017; Oriol et al., 2017), also in the context of Chinese culture (e.g., Wang et al., 2017). Despite these findings, much of the research has focused on a single indicator of school performance (i.e., academic achievement), which yields a limited understanding of the linkage between grit and school adjustment. Moreover, given that students are nested in a complex and interactive environment (Bronfenbrenner, 1979), little is known about how grit may interact with other personal attributes (e.g., social identity) and/or contextual variables (e.g., parental control) to explain the variance of school adjustment.

Although the empirical evidence of the moderating role of grit is still sparse in the literature, some emerging findings may give us some possible indications (e.g., Blalock et al., 2015; Lan and Moscardino, 2019; Lan and Radin, 2020). For example, Lan and Moscardino (2019) found that, in the context of a negative teacher–student relationship, high levels of grit can enhance students’ school well-being (i.e., learning engagement and school satisfaction). The authors explain that gritty students are less impacted by an unfavorable social context (e.g., an unsatisfactory teacher–student relationship) because they focus more on their own long-term determination, therefore resulting in high levels of learning engagement and satisfaction with school. In this perspective, it is conceivable that grit may moderate the association between parental control and school adjustment in Chinese students. To be specific, students with higher levels of grit may buffer the detrimental effect of an unfavorable social context (i.e., high parental control) on school adjustment. Moreover, this association may be more pronounced for students with higher levels of social identity because those who are highly attach to a social group are more easily affected by their parents (Hypothesis 2).

### The Present Study

The objective of the present study was twofold: (a) to investigate the association between parental control and three indicators of school adjustment (social competence, academic grades, and peer acceptance) in a sample of Chinese middle school students; and (b) to examine the moderating role of social identity and grit in this association. According to an ecological framework and empirical findings reviewed above, we generalized two hypotheses:

Hypothesis 1: Parental control is negatively associated with social competence, academic grades, and peer acceptance.
Hypothesis 2: Social identity and grit moderate the association between parental control and school adjustment. Specifically, high social identity may enhance the negative linkage between parental control and school adjustment; moreover, in the context of high social identity, middle school students with a high level of grit may buffer the detrimental effect of parental control on school adjustment (a low level of grit may further exacerbate this detrimental effect).

## Materials and Methods

### Participants

Based on a convenience sampling method, a total of 120 middle school students (42.5% females) aged from 13 to 15 years (*M*_age_ = 13.90; *SD* = 0.70) were involved in the current study. At the time of data collection, participants were attending seventh and eighth grades in public middle schools located in Henan Province, People’s Republic of China. These schools did not use streaming practices, and students were randomly assigned in mixed-ability classrooms when entering the schools. Ninth graders were excluded because these students confronted high academic burdens for entrance examinations during the last year of middle school. In this study, we centered on “typical” middle school students’ school adjustment, and thus we did not set stringent eligibility criteria. There were some general criteria: (a) the students aged from 12 to 15 years were invited to participate in the current study, and this criterion was suggested by a national representative sample of Chinese middle school students (see Cheng et al., 2010); and (b) the participants were full-time students who attended the public middle school in seventh and eighth grades. As a note, 65 students were omitted from the final sample due to the lack of information concerning our study variables (see more details in section “Data Analyses”).

Among these participants, 95.8% belonged to the Han ethnic group, which is the majority ethnic group in China (Ma et al., 2019), to exclude the potential impact of multiethnic effects on students’ school adjustment. In terms of parental educational levels, most fathers (55.8%) and mothers (56.7%) had completed primary or middle school education. With regard to socioeconomic status (SES), 42.5% of participants came from medium-income families (scored from 3 to 5), 31.7% came from high-income families (scored from 6 to 9), and 25.8% came from low-income families (scored from 0 to 2), as measured via the Family Affluence Scale (FAS; Boyce et al., 2006).

### Measures

#### Parental Control

Parental control was measured using the 12-item Chinese Parental Control Scale (CPCS; Shek, 2007), which assesses parental control based on indigenous Chinese concepts. One item example is, “My parents expect me to be mature (*sheng xing*).” Participants were asked to rate each item on a four-point Likert-type scale, ranging from 1 (*strongly disagree*) to 4 (*strongly agree*). The average score of 12 items was calculated, with a higher value indicating greater perception of parental control. Previous study signified good internal consistency of this scale (Shek, 2007). In the current study, Cronbach’s alpha was 0.84. Moreover, Confirmatory Factor Analysis (CFA) was used to ensure the construct validity of this scale in this study. Results showed an acceptable model fit: χ^2^(10) = 20, *p* = 0.02; Tucker Lewis Index (TLI) = 0.90; Comparative Fit Index (CFI) = 0.95; and The Root Mean Square Error of Approximation (RMSEA) = 0.08.

#### Social Identity

Social identity was measured by one subscale of the Collective Self-Esteem Scale (CSE; Luhtanen and Crocker, 1992). This subscale consists of four items. We used CSE to assess social identity, as suggested by prior research (Yip and Cross, 2004, p. 395). More importantly, CSE has been previously validated in Chinese culture (Zhang and Leung, 2002), which can ensure enough statistical power. Participants were asked to consider themselves as members of their important social groups, such as school and family systems. One item example is, “The school/family I belong to is an important reflection of who I am.” Participants were asked to rate each item from 1 (*strongly disagree*) to 6 (*strongly agree*) on a Likert-type scale. The average score of these four items was calculated, with a higher score indicating a higher level of social identity. Prior research revealed the good internal consistency of this scale (Zhang and Leung, 2002). In this study, Cronbach’s alpha was 0.66. Moreover, CFA showed an acceptable model fit of this scale: χ^2^(2) = 5.16, *p* = 0.07; TLI = 0.85; CFI = 0.95; and RMSEA = 0.08.

#### Grit

Grit was measured using one subscale (perseverance, four items) of the Grit Scale (Duckworth and Quinn, 2009), which has been validated in Chinese students by Li et al. (2018). One item example is “Setbacks do not discourage me.” Participants were asked to rate each item ranging from 1 (*not like me at all*) to 5 (*very much like me*) on a Likert-type scale. The average score of these items was calculated, with a higher score indicating a higher level of grit. Based on prior research of Chinese students, this scale showed good internal consistency (Datu and Fong, 2018; Lan and Radin, 2020). In this study, Cronbach’s alpha was 0.67. Moreover, CFA showed an acceptable model fit for this scale: χ^2^(2) = 2.22, *p* = 0.33; TLI = 0.98; CFI = 0.99; and RMSEA = 0.03.

#### Social Competence

Prior research (Renk and Phares, 2004) has suggested that the rating of the social competence of students, as rated by the students themselves, is somehow inaccurate and lacks agreement with other informants. During middle school, Chinese students are intensely involved in school assignments and activities, and teachers have more opportunities to observe their socially appropriate behaviors (Lan et al., 2019c). Given this, teachers are considered as a particularly reliable source of information when reporting their students’ social competence in this context.

In this study, the head teachers in each classroom were asked to finish the four-item subscale of Harter’s Competence Scale for Children (Harter, 1982), which measures school-aged students’ general socially appropriate behaviors. One item example is “This student is usually well-behaved.” Based on a four-point Likert-type scale ranging from 1 (*really false*) to 4 (*really true*), teachers were asked to evaluate students’ social competence. The average score of these items was calculated, with a higher value indicating higher levels of social competence at school. Previous research reported good internal consistency of this scale among Chinese students (Lan et al., 2019c). Cronbach’s alpha was 0.74 in the present study. Moreover, CFA exhibited an acceptable model fit of this scale: χ^2^(2) = 3.20, *p* = 0.20; TLI = 0.97; CFI = 0.99; and RMSEA = 0.07.

#### Academic Grades

Suggested by previous research (Lan et al., 2019c), students’ final exam scores for mathematics, Chinese, and English were gathered from school records at the end of the academic semester. For each subject, the raw test score ranges from 0 to 100, and 60 is considered as a cut-off to pass the exam. Informed by prior research (Lan et al., 2019c), middle school students’ raw test scores were standardized within grades and schools to permit appropriate comparisons. The standardized test scores were used in the further course of analyses, with higher scores indicating better academic performance.

#### Peer Acceptance

Each student was asked to nominate up to three classmates with whom he/she most liked to interact or play, regardless of classmates’ genders. As suggested by a previous study (Chen et al., 2004), the nominations received from all classmates were totaled and then standardized within each classroom to permit appropriate comparisons. The standardized test scores were used in the following analyses, with higher scores indicating greater peer acceptance.

#### Covariates

Age, gender, and SES were treated as control variables because prior research has shown that these variables are potentially related to our dependent variables (e.g., Chen et al., 2019; Lan et al., 2019c). To be precise, demographic information about age and gender was obtained from students’ self-report at the beginning of the survey. Moreover, SES was assessed via the FAS (Boyce et al., 2006), a widely used four-item measure of family wealth (i.e., number of owned cars, number of owned computers, youth’s own bedroom, and frequency of traveling during the past year). FAS has been successfully applied to prior investigation of Chinese students (e.g., Lan et al., 2019a). Scores across items were summed to provide an overall measure of family wealth, with high scores indicating higher levels of family SES.

### Procedure

Before data collection, ethical approval was obtained from Northwest Minzu University. Through personal networks, the research assistant contacted public middle schools in Henan Province. After receiving authorization from school principals, participants (students and their teachers) and the students’ parents were informed about the purposes of this study, and the confidentiality and voluntary nature of this study were strictly guaranteed throughout the research process. These standardized procedures were suggested by well-established empirical research (e.g., Prino et al., 2019; Romera et al., 2019). After the confirmation of written consent from both parents and verbal approval from each student and head teacher, participants were recruited for this study. Overall, the participation rate was 95%, which was in accordance with previous research of Chinese students (Lan, 2019).

During school hours, participants were asked to fill in a set of self-report questionnaires and complete peer nominations within each classroom in 25 min under the supervision of a trained research assistant. In the following 1 or 2 weeks, teachers were asked to fill in the questionnaires to evaluate their students’ social competence at school. In addition, school records concerning academic grades were obtained at the end of this academic term based on each student’s participation IDs. When finishing the participation of this survey, students received a gel pen as a gift to thank for their participation.

### Data Analyses

Data analyses were performed using SPSS 21.0 (IBM Corporation, 2012). Nine cases were eliminated, as those students did not participate in the previous semester’s final exam. Moreover, 56 students from two classrooms were excluded due to the reluctance to participate in sociometric nominations. Overall, 65 students were omitted from the final sample. Additionally, we used a two-step procedure to deal with missing data (Lan and Moscardino, 2019). First, we examined whether there were any students with higher levels of missing data (the cutoff was set at 20% missing values in one or more of the aforementioned scales). In this step, we did not identify any cases, and, thus, no students were excluded from the final sample. Second, to investigate the impact of missing data (less than 20%), a Little’s Missing Completely at Random (MCAR) test was performed. Results supported the MCAR assumption, χ^2^(22) = 27.85, *p* = 0.18. Hence, the remaining missing values were imputed for each student according to the mean score of the corresponding measure.

First, descriptive information for the students was summarized using means and standard deviations for continuous variables. Likewise, Pearson’s correlations were used to evaluate associations among the study variables.

Second, although a structural equation modeling is valuable to address our research questions, the relatively small sample size of this study delimited the possibility of using such an approach, as it would have resulted in too many estimated parameters in relation to the number of study participants (Lan and Moscardino, 2019). Given this, a series of hierarchical multiple regression analyses were conducted to examine the moderating role of social identity and grit in the expected associations between parental control and three indicators of school adjustment.

A hierarchical multiple regression has been widely adopted to evaluate the moderating or interaction effects in psychological research (see a thorough discussion by Petrocelli, 2003). Such an approach is usually conducted to test theoretically based hypotheses. In line with an ecological framework (see the section “Introduction”), we were more interested in testing the interactions—rather than main effects—of parental control on school adjustment. Adopting such an approach would let the predictors enter the regression in a logical order (covariates, main effects, two-way interactions, and three-way interactions). In this context, the focus was on the change in predictability associated with predictor variables entered later in the analysis over and above that contributed by predictor variables entered earlier in the linear regression. The interpretation of the results relied on a change in *R*^2^ and *F* statistics, which were computed by entering the predictors into the analyses at different steps. This would allow researchers to further interpret whether the interactions across multiple contexts instead of the main effects were more informative to the understanding of students’ school adjustment. Given that analyzing possible high-order interactions was the theoretical focus of this paper, we have proposed that using hierarchical multiple regression as a data-analytic strategy would better address our research questions in a more sophisticated and valid manner. Furthermore, it should be noted that the results obtained in the last step of hierarchical multiple regression were the same as those obtained in a single-step multiple linear regression (e.g., using PROCESS macro for SPSS; Hayes, 2013).

In this step of analyses, gender was dummy coded as 1 for males and 2 for females, and all independent variables were centered using the sample mean before creating the interaction terms in the linear equations, as suggested by prior research (Ang and Goh, 2010). In Step 1, the covariates of age, gender, and SES were entered. In step 2, the main effects of parental control, social identity, and grit were entered. Finally, two-way interactions and a three-way interaction were entered in Step 3 and Step 4, respectively. In these analyses, the level of significance was set at *p* < 0.05.

Furthermore, a power analysis was conducted to estimate whether the sample size was appropriate for examining the research questions in this study. Based on an online tool^1^, the results revealed the current sample size could yield a large sample size (>0.35) and adequate statistical power (>0.80).

## Results

### Descriptive Statistics

Descriptive statistics (i.e., means and standard deviations) and bivariate correlations among study variables are reported in Table 1.

**TABLE 1:** Descriptive statistics and bivariate correlations between study variables for Chinese middle school students.

	*M*	*SD*	Range	1	2	3	4	5	6	7	8	9
(1) Parental control	3.35	0.44	2–4	–								
(2) Social competence	2.96	0.50	2–4	0.04	–							
(3) Peer acceptance	0.00	1.00	−9.87–5.87	–0.04	0.44***	–						
(4) Academic grades	0.00	1.00	−1.61–2.81	0.09	0.35***	0.25**	–					
(5) Grit	3.24	0.68	2–5	0.37***	0.13	0.18	0.17	–				
(6) Social identity	4.28	0.99	2–6	0.30***	0.05	0.07	0.12	0.21*	–			
(7) Age	13.90	0.70	13–15	–0.04	–0.05	–0.02	–0.11	–0.01	−0.33***	–		
(8) Gender^a^	–	–	1–2	0.02	0.47***	0.24**	0.19*	–0.07	0.04	−0.24**	–	
(9) SES	4.27	2.23	0–9	0.30***	0.07	0.13	0.08	0.14	0.23**	−0.23**	0.10	–

N = 120. ^*a*^Coded as 1 = male; 2 = female. SES, socioeconomic status. * p < 0.05, ** p < 0.01, *** p < 0.001.

As shown in Table 1, the results indicated that parental control was positively associated with social identity and grit; grit was positively related to social identity. With respect to school adjustment indicators, social competence, academic grades, and peer acceptance were significantly and positively associated with each other. In terms of covariates, age was negatively related to social identity; females were rated by their teachers as more socially competent, nominated by their peers as more highly acceptant, and they achieved a better academic performance compared to their male classmates; and SES was positively related to parental control and social identity.

### Linear Regression Analysis

The results of hierarchical multiple regression analyses are reported in Table 2. In Step 1, the results demonstrated that females showed higher levels of school adjustment performance across three indicators than males. The results of Step 2 illustrated that parental control was negatively associated with academic grades, but grit was positively associated with social competence and academic grades. Moreover, the results of Step 3 showed two significant two-way interaction terms: the interaction between parental control and grit was positively associated with social competence; and the interaction between grit and social identity was negatively associated with social competence. Finally, the results of Step 4 demonstrated two significant three-way interaction terms: the interaction between parental control, grit, and social identity was positively associated with social competence and peer acceptance, respectively. To further understand the nature of these interaction terms, a simple slope analysis was implemented. Since high-order interactions have already included all lower-order interactions for variables in this study, we limited the interpretation to three-way interactions only (Aiken and West, 1991).

**TABLE 2:** The hierarchical multiple regression analyses for three indicators of school adjustment in Chinese middle school students.

	Social competence				Academic grades				Peer acceptance			
	β	*R*^2^	Δ*R*^2^	Δ*F*	β	*R*^2^	Δ*R*^2^	Δ*F*	β	*R*^2^	Δ*R*^2^	Δ*F*
**Step 1**												
Age	0.08				0.06				–0.05			
Gender^a^	0.48***				0.24**				0.18*			
SES	0.03	0.23	0.23	11.38***	0.12	0.08	0.08	3.15*	0.05	0.05	0.05	1.87
**Step 2**												
Age	0.08				0.09				–0.03			
Gender	0.50***				0.26**				0.19*			
SES	0.02				0.14				0.01			
PC	–0.04				−0.19*				0.001			
Grit	0.17*				0.23*				0.17			
SI	0.02	0.26	0.03	1.39	0.06	0.14	0.06	2.62*	0.05	0.08	0.03	1.37
**Step 3**												
Age	0.07				0.08				–0.06			
Gender	0.49***				0.26**				0.19*			
SES	0.01				0.14				–0.001			
PC	–0.05				−0.19*				0.02			
Grit	0.17*				0.23*				0.16			
SI	0.08				0.10				0.06			
PC × Grit	0.24*				0.13				0.18			
PC × SI	–0.03				–0.01				–0.06			
Grit × SI	−21*	0.30	0.04	2.54*	–0.12	0.15	0.01	0.64	–0.01	0.11	0.03	1.28
**Step 4**												
Age	0.07				0.08				–0.06			
Gender	0.49*				0.26**				0.20*			
SES	0.01				0.13				–0.01			
PC	–0.06				−0.20*				0.01			
Grit	0.08				0.21*				0.05			
SI	0.03				0.09				0.001			
PC × Grit	0.27*				0.14				0.22*			
PC × SI	–0.01				–0.01				–0.03			
Grit × SI	−0.30*				–0.14				–0.11			
PC × Grit × SI	0.20*	0.32	0.02	3.27*	0.03	0.15	0.001	0.08	0.25*	0.14	0.03	3.95*

N = 120. ^*a*^Coded as 1 = male, 2 = female. SES, socioeconomic status; PC, parental control; SI, social identity. * p < 0.05, ** p < 0.01, *** p < 0.001.

**FIGURE 1:**
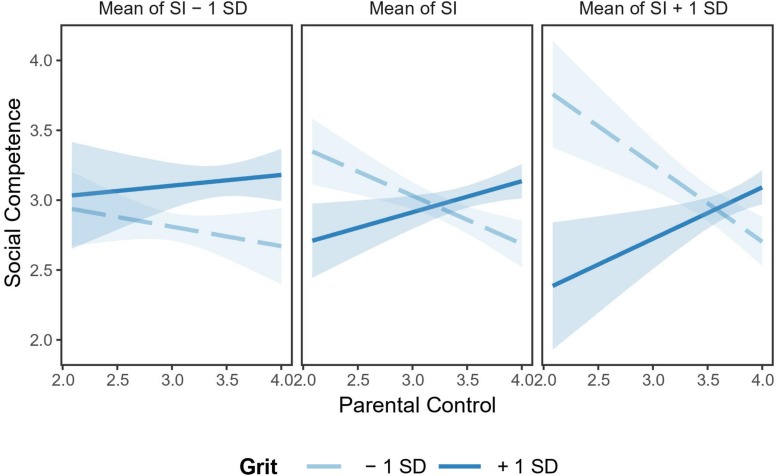
Interaction effect of parental control, social identity, and grit on social competence. Social identity and grit were divided into two levels based on mean: low = *M* – 1*SD*, high = *M* + 1*SD*. *N* = 120. SI, social identity. Light blue bands represent 95% confidence intervals.

The follow-up simple slope analysis illustrated that, when reporting higher levels of social identity, parental control was significantly and negatively related to social competence at lower levels of grit (*B* = −0.55, *SE* = 0.20, *t* = −2.82, *p* < 0.01) but not at higher levels of grit (*B* = 0.37, *SE* = 0.21, *t* = 1.74, *p* = 0.09). By contrast, when reporting lower levels of social identity, the association between parental control and social competence was not significant at both higher levels of grit (*B* = 0.08, *SE* = 0.21, *t* = 0.37, *p* = 0.71) and lower levels of grit (*B* = −0.14, *SE* = 0.20, *t* = −0.69, *p* = 0.49; see Figure 1).

**FIGURE 2:**
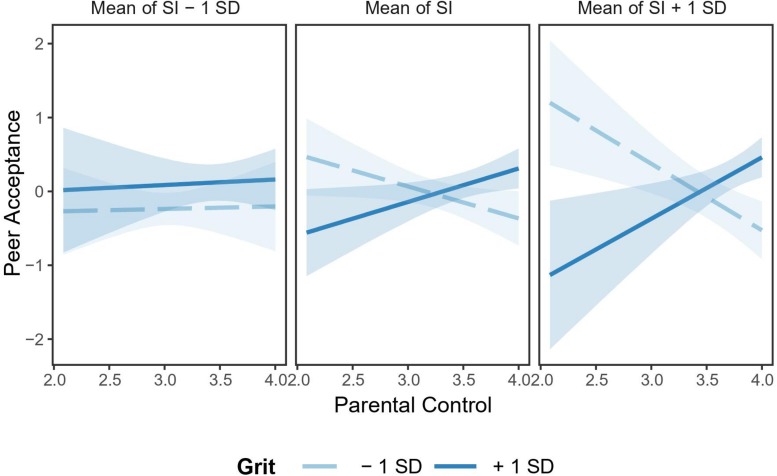
Interaction effect of parental control, social identity, and grit on peer acceptance. Social identity and grit were divided into two levels based on mean: low = *M* – 1*SD*, high = *M* + 1*SD*. *N* = 120. SI, social identity. Light blue bands represent 95% confidence intervals.

Furthermore, when reporting higher levels of social identity, parental control was significantly and negatively linked to peer acceptance at lower levels of grit (*B* = −0.90, *SE* = 0.43, *t* = −2.09, *p* < 0.05) but not at higher levels of grit (*B* = 0.83, *SE* = 0.47, *t* = 1.77, *p* = 0.08). In contrast, when reporting lower levels of social identity, the association between parental control and peer acceptance was not significant at both higher levels of grit (*B* = 0.07, *SE* = 0.46, *t* = 0.16, *p* = 0.87) and lower levels of grit (*B* = 0.03, *SE* = 0.45, *t* = 0.08, *p* = 0.94; see Figure 2).

## Discussion

Although the association between parental control and school adjustment has been extensively addressed in many cultural contexts, findings of this association are less clear-cut. Moreover, little is known about this association in middle school students. Guided by an ecological framework, the current study examined the association between parental control and three objective indicators of school adjustment among Chinese middle school students. Furthermore, the moderating role of social identity and grit on this association was ascertained. The results showed that parental control was negatively associated with academic grades; and social identity and grit moderated the associations between parental control and social competence as well as between parental control and peer acceptance. Specifically, when reporting higher levels of social identity, parental control was negatively associated with social competence and peer acceptance for those students with lower levels of grit.

Our first objective was to investigate the association between parental control and three indicators of school adjustment. In line with the first hypothesis, the results showed that parental control was negatively associated with academic grades among middle school students. Such a finding is consistent with prior research indicating that parental control has a detrimental effect on students’ academic achievement (Kokkinos and Vlavianou, 2019). This is probably because parental control forces students to focus more on internal distress and parental approval, rather than the learning process itself, which further hinders students’ autonomous motivation and academic performance. Another possible explanation is attributed to the developmental nature of this period. During middle school, the rate of asking for autonomy and independence dramatically increases due to the cognitive and biological development of students (Roche et al., 2014). Meanwhile, academic stress also increases due to the imminent approach of high school entrance examinations (Cheng et al., 2010). Under these salient stressors, high parental control can dampen students’ learning interests and motivations, which in turn restrains their academic performance.

With regard to covariates, the results showed that females outperformed males in terms of three school adjustment indicators. Such a finding is in accordance with prior research into school-aged children (Driessen and Van Langen, 2013; Fischer et al., 2013). These results can be interpreted with regard to gender differences in cognitive and non-cognitive competencies. As documented by prior research (Driessen and Van Langen, 2013), female students did better than males in reading and language. In this study, the obtained academic grades were mainly derived from language test performance (i.e., Chinese and English). Thus, females may have had a slight advantage in terms of academic grades than males. Moreover, in middle school, males are more often engaged in conflicts (Driessen and Van Langen, 2013); in contrast, influenced by traditional gender identity, females show better self-control and perform socially appropriate behaviors and adequate social skills.

Moreover, in line with the second hypothesis, we found significant three-way interactions in terms of peer acceptance and social competence. Specifically, in the context of high social identity, the negative associations between parental control and social competence, as well as between parental control and peer acceptance, were significant at lower levels of grit (as opposed to higher levels of grit). One possible explanation is that students with high social identity are likely to regard themselves as closely connected with their family, and the detrimental effect of parental control on school adjustment can be amplified (to some extent). Such a manner may exacerbate their ability to regulate emotions and develop appropriate coping strategies, thereby resulting in inappropriate social interactions (McDowell and Parke, 2005). Furthermore, this association was more pronounced in terms of lower levels of grit. This may be because less perseverance is not culturally favored (Li et al., 2018). Thus, low grit can further exacerbate the detrimental effect of parental control on social competence and peer acceptance in middle school students. Less gritty students are more affected by an unfavorable social context (i.e., parental control), and lack of grit hinders their capacity to focus on own long-term determination, thereby leading to students’ negative social competence and peer acceptance.

However, the three-way interaction was not significant regarding academic grades. One possible explanation is that the possible interaction effect is, to some extent, hindered by the strong direct associations between parental control and academic grades and between grit and academic grades. Such an explanation is in line with previous findings. For instance, in many contextual contexts, parental control is directly related to students’ academic grades (Pinquart, 2016; Pinquart and Kauser, 2018). As for Chinese parents, they are involved more in their children’s academic assignments because of the long-standing cultural linkage between academic achievement and family dignity. Likewise, as documented by many studies (Muenks et al., 2017; Oriol et al., 2017; Wang et al., 2017), individuals’ perseverance is directly linked to their academic grades. As a note, this insignificant interaction may also be due to the relatively small sample size and several limitations, as we discussed below. Thus, researchers should keep cautious when interpreting these findings.

### Limitations

Along with these significant findings, several limitations should be acknowledged. First, the current study was built on a cross-sectional design, which has less power than a longitudinal design when it comes to excluding time-invariants and unobserved individual differences as well as in terms of observing a certain event’s temporal order (Lan and Wang, 2020). Although much of the research encompasses parental influence as a predictor of the school performance of their respective children (Pinquart, 2016), there is a possibility that a low academic achievement may disturb positive parent–child relationships. Future studies should use a longitudinal design to ascertain this issue and further demonstrate how social identity and grit interacting with parental control to illustrate the developmental trend of school adjustment among students.

Second, the current study does not differentiate maternal and paternal control. As indicated by previous investigations in many cultural contexts (e.g., Laible and Carlo, 2004; Lan et al., 2019c), maternal—but not paternal control—is strongly related to students’ adjustment at school. Likewise, maternal and paternal control are distinctly associated with students’ adjustment across different domains (Cascio et al., 2016). Given this, future initiatives are recommended to address these concerns and further develop a more fine-grained understanding of the association between parental control and school adjustment.

Relatedly, this study has failed to differentiate between the multiple forms of parental control (i.e., psychological and behavioral control; see Grolnick and Pomerantz, 2009, for a review). Empirical studies have shown that behavioral control is positively correlated with academic achievement, whereas psychological control is negatively related to academic achievement (e.g., Kokkinos and Vlavianou, 2019). Given that these two types of parental control are somewhat related but distinct from each other, it is critical to distinguish the unique association between each form of parental control and school adjustment.

Third, although the research measurements rely on some methodologically sound and well-validated tools in the Chinese culture, the internal consistency coefficients of social identity and grit scales are notably lower than those reported by Zhang and Leung (2002) and Datu and Fong (2018). One possible explanation is ascribed to the small sample size in relation to the number of items in this study. In this regard, future studies utilizing a larger sample size may consider replicating the current findings in order to increase the research reliability. Moreover, simplified wording on several items in these scales should be considered, as middle school students may have difficulty with understanding some of these items.

Finally, as mentioned above, the association of parental control with school adjustment may vary, to some extent, between different cultural contexts (e.g., ethnic groups or regions; Pinquart and Kauser, 2018). Thus, this investigated association is interpreted differently, relying on the cultural meaning of parental control in the particular cultural group. Given the salience of this issue in family relations and child development, further investigation is highly recommended to conduct a cross-cultural design and to generalize these research questions into more broad populations and cultural contexts.

### Contributions and Implications

Despite these visible limitations, the present research may contribute to the literature in many ways. First, the research data were gathered from multiple resources, which may provide a more holistic picture of students’ school adjustment. Second, the current study has expanded upon the existing literature by investigating the association between parental control and a variety of indicators of school adjustment in middle school students, who have been shown to encounter several psychosocial and academic challenges. Third, based on a robust theoretical framework, we have added to the growing literature by documenting the moderating role of social identity and grit in the parenting-adjustment linkage. Such an investigation can provide some novel insights into designing school activities because the malleability of these individual characteristics enables educators and practitioners to incorporate them into school-based activities. Finally, although the present research is built on a single culture context, we have proposed that the investigation of parental control and school adjustment in the Chinese society may provide a solid foundation for scientific studies targeting family relations in other cultural contexts. With the rapid economic growth and fierce competition in occupational admission, we believe that researchers from Western societies are also striving to understand how to regulate family relations better and foster students’ school adjustment successfully. Given the salience of school adjustment in students, it is conceivable that successful school adjustment during this period may have a positive long-term effect on the future professional careers of the students, their attainment of social status, as well as of their psychological well-being.

Moreover, based on these significant findings, several theoretical and practical implications can be made. Regarding theory, the present study enriches the ecological framework (Bronfenbrenner, 1979) and pertinent literature to the correlates of students’ school adjustment. To be specific, although the global importance of parenting styles has been acknowledged, researchers should realize that family processes always occur within a complex context. Thus, family processes may have different effects on different groups of individuals. In a sense, to have a more fine-grained understanding of students’ school adjustment, researchers should realize that students are embedded in an ecological context, and multiple settings interactively shape their school adjustment.

From a practical perspective, although traditional Chinese culture expects children to obey their parents’ rules and achieve high academic grades (Shek, 2007), this parenting style should be adjusted in the modern society. For example, parents should provide a meaningful rationale, acknowledge negative feelings, use non-controlling language, offer meaningful choices, and nurture internal motivational resources in their children (Vansteenkiste et al., 2005; Lan et al., 2019a). Moreover, school-based activities should focus on enhancing social identity and grit, which facilitates students’ social interaction at school. For instance, collaborative or cooperative school activities should be regularly organized to foster students’ social identity within their own classroom. Meanwhile, long-term goals nested in these activities should be established (Lan and Moscardino, 2019), and educators or practitioners should inspire students to practice when approaching these goals.

## Conclusion

We have endeavored to integrate the conflicting findings of the association between parental control and school adjustment into a coherent body of knowledge, paying particular attention to the moderating role of social identity and grit within the context of middle school students, with a broader implication for educators and practitioners. Gathering information from multiple resources, the current study indicates that parental control is negatively linked to academic grades in middle school students. Moreover, low levels of grit can magnify the detrimental effect of parental control on social competence and peer acceptance in middle school students who regard themselves as closely connected to social groups.

## Data Availability Statement

The datasets generated for this study are available on request to the corresponding author.

## Ethics Statement

The studies involving human participants were reviewed and approved by Northwest Minzu University. Written informed consent to participate in this study was provided by the participants’ legal guardian/next of kin.

## Author Contributions

CM conceived and drafted the manuscript. YM was one of the principal investigators of this study. XL performed the statistical analyses and critically revised the manuscript. All authors read and approved the final draft of the manuscript.

## Conflict of Interest

The authors declare that the research was conducted in the absence of any commercial or financial relationships that could be construed as a potential conflict of interest.

## Footnotes

1 https://www.danielsoper.com/statcalc/calculator.aspx?id=1
